# Allosterically Linked Binding Sites in Serotonin Transporter Revealed by Single Molecule Force Spectroscopy

**DOI:** 10.3389/fmolb.2020.00099

**Published:** 2020-06-03

**Authors:** Rong Zhu, Walter Sandtner, Joan E. A. Ahiable, Amy Hauck Newman, Michael Freissmuth, Harald H. Sitte, Peter Hinterdorfer

**Affiliations:** ^1^Institute of Biophysics, Johannes Kepler University Linz, Linz, Austria; ^2^Institute of Pharmacology and the Gaston H. Glock Research Laboratories for Exploratory Drug Development, Center of Physiology and Pharmacology, Medical University of Vienna, Vienna, Austria; ^3^Medicinal Chemistry Section, Molecular Targets and Medications Discovery Branch, National Institute on Drug Abuse, Intramural Research Program, Baltimore, MD, United States

**Keywords:** serotonin transporter, S-citalopram, allosteric binding sites, atomic force microscopy, single molecule force spectroscopy, simultaneous topography and recognition imaging

## Abstract

Crystal structures and experiments relying on the tools of molecular pharmacology reported conflicting results on ligand binding sites in neurotransmitter/sodium symporters (NSS). We explored the number and functionality of ligand binding sites of NSS in a physiological setting by designing novel tools for atomic force microscopy (AFM). These allow for directly measuring the interaction forces between the serotonin transporter (SERT) and the antidepressant S-citalopram (S-CIT) on the single molecule level: the AFM cantilever tips were functionalized with S-CIT via a flexible polyethylene glycol (PEG) linker. The tip chemistry was validated by specific force measurements and recognition imaging on CHO cells. Two distinct populations of characteristic binding strengths of S-CIT binding to SERT were revealed in Na^+^-containing buffer. In contrast, in Li^+^-containing buffer, SERT showed only low force interactions. Conversely, the vestibular mutant SERT-G402H merely displayed the high force population. These observations provide physical evidence for the existence of two binding sites in SERT. The dissociation rate constant of both binding sites was extracted by varying the dynamics of the force-probing experiments. Competition experiments revealed that the two sites are allosterically coupled and exert reciprocal modulation.

## Introduction

The serotonin transporter (SERT) is a member of the monoamine neurotransmitter sodium symporter (NSS) family (Kristensen et al., 2011). Its eponymous action is to retrieve extracellular serotonin into the presynaptic specialization or into platelets. In the brain, released serotonin acts predominantly as a neuromodulator; the action of SERT is to limit volume transmission and replenish presynaptic vesicular stores. A number of neurological conditions such as depression, anxiety disorder, attention-deficit hyperactivity disorder (ADHD), autism, psychostimulant use disorders, and Parkinson’s disease are related to NSS dysregulation. SERT is a target for medications such as S-citalopram (S-CIT) to treat anxiety and depression. However, illicit drugs such as cocaine and MDMA “ecstasy” also bind to SERT. Some X-ray analysis of SERT (Coleman and Gouaux, 2018; Coleman et al., 2019) and its homologs, i.e., bacterial LeuT_Aa_ (Yamashita et al., 2005) and dDAT (the *Drosophila melanogaster* dopamine transporter) (Penmatsa et al., 2013; Wang et al., 2015), indicate a primary binding site (S1-site) for substrates in the membrane-spanning region. Other studies provided hint for a second S2-site, located within the extracellular vestibule (Plenge and Mellerup, 1997; Sarker et al., 2010; Plenge et al., 2012; Coleman et al., 2016) of NSS transporters. To elucidate the number and the mechanism of ligand binding site(s) in SERT, single molecule force spectroscopy (SMFS) was used to directly measure the interaction forces between SERT and S-CIT (Zhu et al., 2015).

## Coupling of Antidepressant Drug to AFM Tips

**FIGURE 1:**
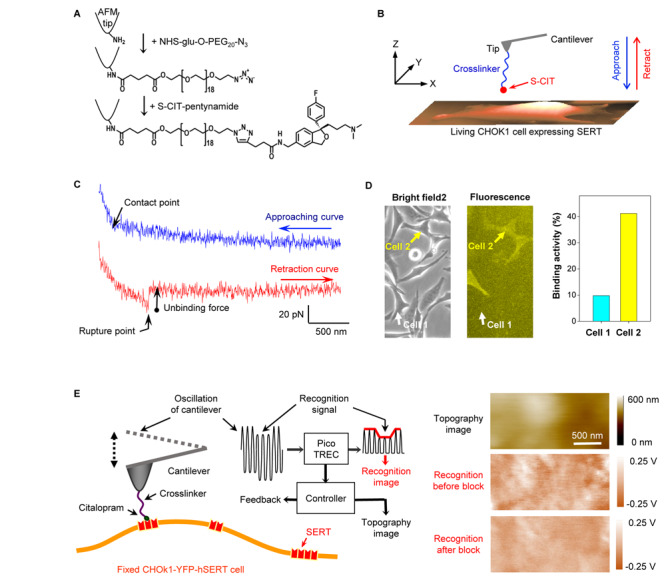
**(A)** Conjugation of S-CIT to AFM cantilever tip. After the cantilever was amino-functionalized in the gas phase APTES (Riener et al., 2003), it was pegylated with NHS-glu-O-PEG_20_-N_3_ (Zhu et al., 2015). Thereafter, the alkyne-modified S-CIT analog was coupled to the azido-terminated PEG via co-catalyst-accelerated copper(I)-catalyzed azide-alkyne cycloaddition (Lewis et al., 2004). **(B)** The S-CIT adorned cantilever tip was used to record force curves **(C)** on living CHOK1 cells expressing human SERT fused with YFP. **(D)** Cells seeded on the same dish showed different expression levels of SERT. The binding activity increased with the expression level, suggesting that the binding events arose from specific interactions between tip-coupled S-CIT and SERT. **(E)** Recognition imaging (principle shown in left part) revealed nano domains of SERT (shown as dark spots in the recognition image) in the cell membrane with diameters around 100–200 nm. After adding free CIT into the solution, the interaction between SERT and CIT-adorned AFM tip was blocked, resulting in the disappearance of the recognition spots (recognition image after block). **(A–C)** are reproduced from Zhu et al. (2015, 2018) with permission.

To date, a series of crosslinkers have been developed for covalent conjugation of functional molecules to atomic force microscopy (AFM) cantilever tips. Some conventional linkers are NHS-PEG-aldehyde (Ebner et al., 2007), NHS-PEG-acetal (Wildling et al., 2011), NHS-PEG-PDP (Haselgruebler et al., 1995), and NHS-PEG-maleimide (Neundlinger et al., 2014). For AFM experiments, the 5-aminomethyl analog (Kumar et al., 2014) of pure S-CIT was synthesized. Although this molecule can be directly linked to the AFM tip by using NHS-PEG-aldehyde or NHS-PEG-acetal linker, we were concerned that the positive charge of the amine group in the short linker of 5-aminomethyl-S-CIT may interfere with specific molecular recognition. Therefore, this molecule was further modified with an alkyne group, for covalent conjugation to the AFM cantilever tip with NHS-PEG-azide linker via click chemistry, as shown in Figure 1A (Zhu et al., 2015). The validity of the tip chemistry was shown by both single-molecule force spectroscopy and recognition imaging.

Force measurements were conducted on living CHOK1 cells (Figure 1B) stably expressing human SERT (Wildling et al., 2012) fused with yellow fluorescence protein (YFP) in HEPES buffer. The S-CIT-adorned AFM cantilever tip was lowered toward the cellular surface to allow for binding to SERT, before it was subsequently moved upward. Because the S-CIT moiety on the AFM tip established a bond to SERT on the cell surface, a pulling force developed during the upward movement between the tip and cell membrane, causing the cantilever to bend downward (Figure 1C). The pulling force increased up to a critical value during further pulling until the bond between SERT and S-CIT was ruptured (unbinding force). In order to examine whether the binding events between SERT and S-CIT were specific, the same AFM tip was used to measure cells with different expression level of SERT visualized by fluorescence microscopy (Figure 1D). Force curves were repeatedly recorded using the same conditions. The binding activity, which is defined as the percentage of force curves showing binding events, increased correspondingly with the expression level of SERT. This indicated that the binding events were indeed specific.

The validity of the tip chemistry was also shown by topography and recognition imaging (TREC) (Figure 1E). TREC is a label-free super-resolution imaging technique (Stroh C. et al., 2004; Stroh C. M. et al., 2004). For simultaneously recording a topography and recognition image, the cantilever was oscillated at constant amplitude (Figure 1E) while laterally scanning the surface of a cell, which was fixed with 4% paraformaldehyde for 15 min. The electronic circuits in the Pico-TREC box split the oscillation signal into upper and lower parts (Figure 1E, left panel), from which the lower oscillation parts (downstrokes) yielded the topographical image (height surface relief, see Figure 1E, right panel). Whenever the CIT on the AFM tip bound with SERT in the cell membrane, the upward oscillations of the cantilever were reduced due to the physical connection between tip and cellular surface. From the envelope of the upper part of the oscillations, the recognition image was constructed (Figure 1E, right panel). The location of the binding event was presented as dark spots in the recognition image. Subsequently, free CIT was added into the buffer solution with a final concentration of 0.09 mM for blocking the interaction between the SERT and the CIT on the AFM tip. Consequently, the recognition spots in the recognition image disappeared, indicating that the recognition indeed arose from the specific binding of CIT and SERT (Figure 1E, right panel).

## Two Binding Sites in Sert

**FIGURE 2:**
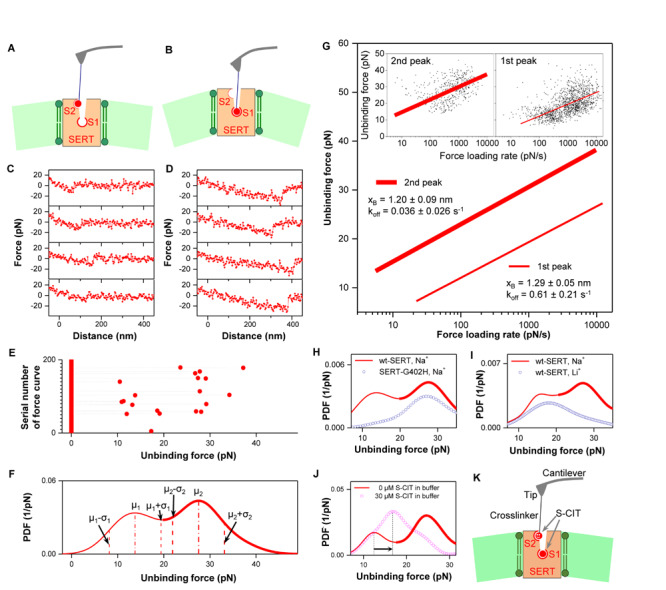
**(A–E)** Two populations of interaction forces displaying different force strength were observed from repeated force measurements (Zhu et al., 2015). Four representative curves of each population are shown in **(C)** and **(D)**, respectively, which arose from two different binding sites **(A,B)** in SERT. Larger forces result in longer unbinding lengths that mainly arise from deformation of the elastic cell membrane. From repeatedly recorded data **(E)**, an experimental probability density function (PDF) of the unbinding forces was generated **(F)**. For analysis of each individual binding site, the unbinding events were separated by using the range μ–σ to μ + σ, where μ is the center of the peak and σ is the standard deviation of the Gaussian fitting (Zhu et al., 2018). **(G)** The data points of unbinding forces for the S1 (2nd peak) and S2 (1st peak) binding site, respectively, were plotted against the logarithm of the force loading rate (insets). They were fitted with Evans’ single energy barrier model, from which the kinetic off rate k_off_ and the width of the energy barrier x_B_ were extracted. **(H)** Force measurements on mutant SERT-G402H (point mutation in vestibular S2 site) show only a single peak, which corresponds to the second peak for wt SERT. This indicates that the S2 binding site is not accessible with the mutation, but the S1 site remains intact. **(I)** Force measurements on wt SERT in buffer without Na^+^ (Li^+^ buffer) display only a single peak as well, which corresponds to the first peak for wt SERT in Na^+^ buffer. This indicates that the S1 site is completely inactivated in Li^+^ buffer, but the S2 binding site is still active. **(J)** 0.03 mM S-CIT in solution blocked the S1 site (the second peak in the force PDF). The S2 site was slightly strengthened, as shown by the shift of the first peak in force PDF to the right, indicating a positive allosteric effect **(K)**. **(A–I)** are reproduced from Zhu et al. (2015, 2018) with permission.

From a large amount of repeated force measurements using the same conditions, two populations with distinct unbinding forces (Figures 2A–E) were observed. For each unbinding force value, a Gaussian of unitary area with its center representing the unbinding force and the width (standard deviation) reflecting its measuring uncertainty (square root of the variance of the noise in the force curve) was computed. All Gaussians from one experimental setting were accordingly summed up and normalized with its binding activity to yield the experimental probability density function (PDF) of unbinding force (Figure 2F). The PDF represents the original data and can be viewed as the equivalent of continuous histograms. The PDF of unbinding forces obtained from an S-CIT-modified tip (Figure 2F) showed two distinct peaks, most likely reflecting S-CIT rupture from SERT binding sites with two different strengths of interaction.

To identify the location of the binding sites reflecting the two force populations, force measurements were performed for S-CIT on SERT-G402H, which contains a point mutation in the outer vestibule (Plenge et al., 2012). The force PDF from SERT-G402H (Figure 2H, dotted curve) showed only a single peak, which corresponded to the second peak in the force PDF from wild type (wt) SERT. This indicates that the first peak in the force PDF from wt SERT originates from the vestibular S2 site, which is not accessible in SERT-G402H, whereas the stronger binding site (S1) of SERT-G402H remained intact. With the G402H substitution, the 5-[^3^H]HT uptake was not detectable (Table 1 in Plenge et al., 2012). However, S-CIT can still bind at S1 site with IC_50_ value of 8.9 nM, which is only slightly changed from 2.8 nM for the wt (Table 1 in Plenge et al., 2012). At the S2 site, the binding affinity of S-CIT is drastically changed from 4.6 μM to >1000 μM (Table 3 in Plenge et al., 2012). Although the amino acid residue G402 is not involed in the binding of S-CIT to S2 site, the mutation G402H might provide the steric hinderance or possibly induce the structural rearrangement, thus disrupting the S2 site. In addition, it was verified that Na^+^ ions are important for accessibility to the SERT binding sites by recording forces of S-CIT binding to wt SERT in Li^+^ buffer, where NaCl was replaced by Li_2_CO_3_. The PDF showed only a single peak in the force PDF (Figure 2I, dotted curve) that coincided with the first peak in the force PDF observed in Na^+^ buffer (Figure 2I, solid curve). The absence of the second peak is consistent with the notion that the central S1 site is Na^+^-dependent. The persistence of the first peak implies that access to the vestibule S2 site does not require Na^+^.

We extracted the dissociation rate constant (k_off_) and the width (x_B_) of prominent energy barriers of the interaction for the individual force populations by plotting the unbinding forces as a function of the force loading rate (equivalent to pulling velocity times effective spring constant during pulling) for both S1 and S2 (Figure 2G). We relied on a maximum likelihood approach to fit the experimental data to the statistical model: the Evans model (Evans, 1999) was employed as an analytical model to estimate the maximum likelihood of x_B_ and k_off_. We verified the results from the force spectroscopy experiments by measuring k_off_ of pegylated S-CIT by electrophysiological recordings in the whole-cell patch-clamp configuration. The k_off_ values of pegylated S-CIT (0.064 ± 0.010 s^–1^) were in agreement with those determined for the S1 site by force spectroscopy (0.032 ± 0.005 s^–1^) (Zhu et al., 2015).

## Allosteric Effect From Central S1 Site to Vestibular S2 Site

Dissociation of S-CIT at the central S1 site is delayed, if the vestibular S2 site is occupied by either S- or R-CIT (Plenge et al., 2012). This observation was interpreted as an allosteric modulation – via steric hindrance – of the S1 site by the S2 site. In the force spectroscopy study, the interplay between the S1 and S2 sites was explored by performing competition experiments with free S-CIT at saturating conditions (0.03 mM), in which the higher affinity central S1 binding site was occupied whereas the lower affinity vestibule binding site was still accessible to S-CIT on the tip. In the presence of S-CIT in solution only a single peak remained in the force PDF (Figure 2J, dotted curve), which was attributed to the vestibule S2 site. This can be accounted for by the long dwell time (∼20 min) of S-CIT at the S1 site (Plenge and Mellerup, 1997), which blocked the possibility for the S-CIT on the AFM tip to bind with the S1 site in the SERT. However, the AFM-tip tethered S-CIT, which has a local concentration of ∼3.7 mM (corresponding to one S-CIT molecule within a hemisphere of radius ∼6 nm, defined by the length of PEG linker) (Kamruzzahan et al., 2006), is likely to effectively compete with the S-CIT in solution (0.03 mM) for binding to the S2 site. The position of the maximum of the remaining peak in the force PDF was shifted to the right when compared to the interaction forces of the S2 site in the absence of S-CIT in the solution (arrows in Figure 2J). This result indicates that, when S-CIT binds at the central S1 site, it exerts a positive allosteric effect on the vestibular S2 site, i.e., enhances the binding of S-CIT at the S2 site (Figures 2J,K).

## Discussion

The flexible PEG linker between the AFM tip and the CIT molecule provides the unique opportunity to explore the binding sites of different depths in transmembrane transporter. Force sensing in physiological conditions allowed for the extraction of dynamic information, thus unequivocally documenting the existence of two binding sites in SERT. Furthermore, our force experiments revealed a positive allosteric effect on the S2 site induced by the binding of S-CIT at S1 site. Given the importance of allosteric regulation in biology and pharmacology, SMFS provides a unique approach to explore transient binding sites in clinically relevant membrane transporters for addressing the modulation of interaction forces between ligands and allosterically coupled binding sites.

Indeed, such an allosterically enhanced binding strength of S-CIT at the S2 site could be a critical contribution for the capture of the S2 site by X-ray crystallography. The X-ray structure of SERT bound with S-CIT (Coleman et al., 2016) verified the two binding sites in SERT. We notice that crystal structures (Coleman et al., 2016) and earlier experiments (Plenge et al., 2012) reported different S2 sites for S-CIT binding to the SERT. In the earlier experiments using a series of SERT mutants (Plenge et al., 2012), the S2 site was found to be surrounded by the amino acid residues L99, W103, R104, I179, V236, L237, G402, A486, and V489. In a later study with bivalent ligands (Andersen et al., 2016), the S2 site was found at very similar position surrounded by the amino acid residues I179, D400, P403, V489, and K490. However, in the crystal structure (Coleman et al., 2016), the S2 site is surrounded by the amino acid residues R104, D328, A331, Q332, E494, F556, and P561. It was found that the most pronounced decrease in allosteric potency occurred after substitution of the EL4a-EL4b linker residue G402 by histidine (Plenge et al., 2012), which resulted in elimination of the allosteric action of S-CIT. It is noticed (Cheng and Bahar, 2019) that this residue is sequentially adjacent to LeuT residue F320, which triggers allosteric responses in LeuT (Cheng and Bahar, 2013). These independent studies identified the critical residue in the same region. Moreover, all MATs have a conserved glycine at this position, indicative of their functional importance. Nevertheless, the distance between G402 and the S-CIT at the S2 site in the crystal structures (PDB: 5173, 5175) (Coleman et al., 2016) is at least 0.7 nm. The difference might be caused by two possibilities. One possibility is that the mutation changed the structural arrangement, i.e., even the amino acid residue (for example, G402) is not involved in the binding of S-CIT to S2 site, but the mutation (e.g., G402H) induced the steric hindrance or structural change thus disrupting the S2 site. The other possibility is that the process of crystallization or freezing might slightly change the structure of the protein and the position of ligand in the protein. Currently, there is no enough evidence to confirm either hypothesis. This problem might be solved by NMR structure or other techniques which obtain the structural information in physiological conditions.

We notice that the only common amino acid residue of S2 site revealed by the crystal structure and previous studies is the arginine R104. The involvement of R104 in S2 site is partially supported by the experiment of recognition imaging (Figure 1E). The block of the binding sites in the AFM experiments is mainly determined by the lifetime of the bond. At S1 site, S-CIT can stay there for more than 10 min. Therefore, with 0.03 or 0.09 mM free S-CIT in the buffer solution, S1 site can be stably blocked. However, the lifetime of the bond at S2 site is only about 1 s. Therefore, the AFM-tip tethered S-CIT, which has a local concentration of ∼3.7 mM, can effectively compete with the S-CIT in solution (0.03 or 0.09 mM) for binding to the S2 site of the SERT in the living CHOK1 cell. For recognition imaging (Figure 1E), the cells were fixed with paraformaldehyde. Because the S1 site of the SERT has no lysine or arginine residue (Coleman et al., 2016, 2019; Coleman and Gouaux, 2018), after the fixation of the cell the S1 site of the SERT still remains intact. However, the S2 site of the SERT contains an arginine residue R104 (Plenge et al., 2012; Coleman et al., 2016) and a lysine residue K490 (Plenge et al., 2012; Andersen et al., 2016), after the fixation of the cell the S2 site of the SERT is already blocked by the fixative. Therefore, the free CIT in the solution completely blocked the binding on the fixed cells in the recognition imaging.

Further developed AFM techniques have provided the possibility to measure the depth of ATP binding site in mitochondrial uncoupling protein 1 (UCP1) (Zhu et al., 2013) or to reconstruct three-dimensional image of chemical groups inside a protein complex (Kim and Sahin, 2015). Since the S2 site in crystal structure (Coleman et al., 2016) is more than 0.5 nm deeper than that determined by other experiments (Plenge et al., 2012), the measurement of the depth of the S2 site using these promising AFM techniques should eventually clarify the controversy of the position of S2 site.

## Author Contributions

All authors listed have made a substantial, direct and intellectual contribution to the work, and approved it for publication.

## Conflict of Interest

The authors declare that the research was conducted in the absence of any commercial or financial relationships that could be construed as a potential conflict of interest.
